# Race, Ethnicity, and Sex Among Senior Faculty in Radiation Oncology From 2000 to 2019

**DOI:** 10.1001/jamanetworkopen.2021.42720

**Published:** 2022-01-11

**Authors:** James R. Janopaul-Naylor, Sanford E. Roberts, Hui-Kuo Shu, Aparna H. Kesarwala, Jolinta Y. Lin, Jeffrey M. Switchenko, Mylin A. Torres

**Affiliations:** 1 Department of Radiation Oncology, Winship Cancer Institute, Emory University, Atlanta, Georgia; 2 Department of General Surgery, Perelman School of Medicine, University of Pennsylvania, Philadelphia; 3 Department of Biostatistics and Bioinformatics, Rollins School of Public Health, Emory University, Atlanta, Georgia

## Abstract

This cross-sectional study investigates intersections among race, ethnicity, and sex from 2000 to 2019 among senior faculty in radiation oncology.

## Introduction

Radiation oncologists provide care to approximately 50% of patients with cancer.^1^ However, the proportion of medical students who are women or members of underrepresented minority groups who pursue radiation oncology training is disproportionately low even compared with other oncologic specialties.^2^ While senior faculty play a critical role in training future physicians, no study has examined senior faculty demographics in radiation oncology, to our knowledge. This study evaluated intersections of race, ethnicity, and sex over time among senior faculty in radiation oncology compared with other departments.

## Methods

Approval and informed consent for this cross-sectional study were waived by the Emory University Institutional Review Board given that this study was not a clinical investigation with identifiable human participants. The Strengthening the Reporting of Observational Studies in Epidemiology (STROBE) reporting guideline was followed. We requested Association of American Medical Colleges 2000 to 2019 data on self-reported sex, race, ethnicity, and faculty rank of academic physicians. Race and ethnicity were assessed to better understand academic physician disparities not accurately depicted by sex, race, or ethnicity alone. We developed cohorts based on intersections of sex, race, and ethnicity (eg, non-Hispanic Black women) and then determined proportions of senior faculty (ie, associate or full professors) in each group. Black, Hispanic, American Indian, Alaska Native, Native Hawaiian, and other Pacific Islander individuals were considered members of underrepresented minority groups. The other race and ethnicity categories that were available included White, Asian, other, multiple race–Hispanic, multiple race–non-Hispanic, and unknown. We assessed changes in representation over time using Cochran-Armitage trend tests. Trends in radiology, internal medicine, and general surgery were also examined, given that they are popular fields that contribute to the oncology workforce. All *P* values were 2-sided, unless otherwise noted. Statistical significance was assessed at the *P* < .05 level. Multivariable linear regression models were fit, incorporating year, medical specialty, and interaction of year and specialty. Statistical analysis was conducted using SAS statistical software version 9.4 (SAS Institute). Data were analyzed from August 2020 through July 2021.

## Results

**Figure 1.**
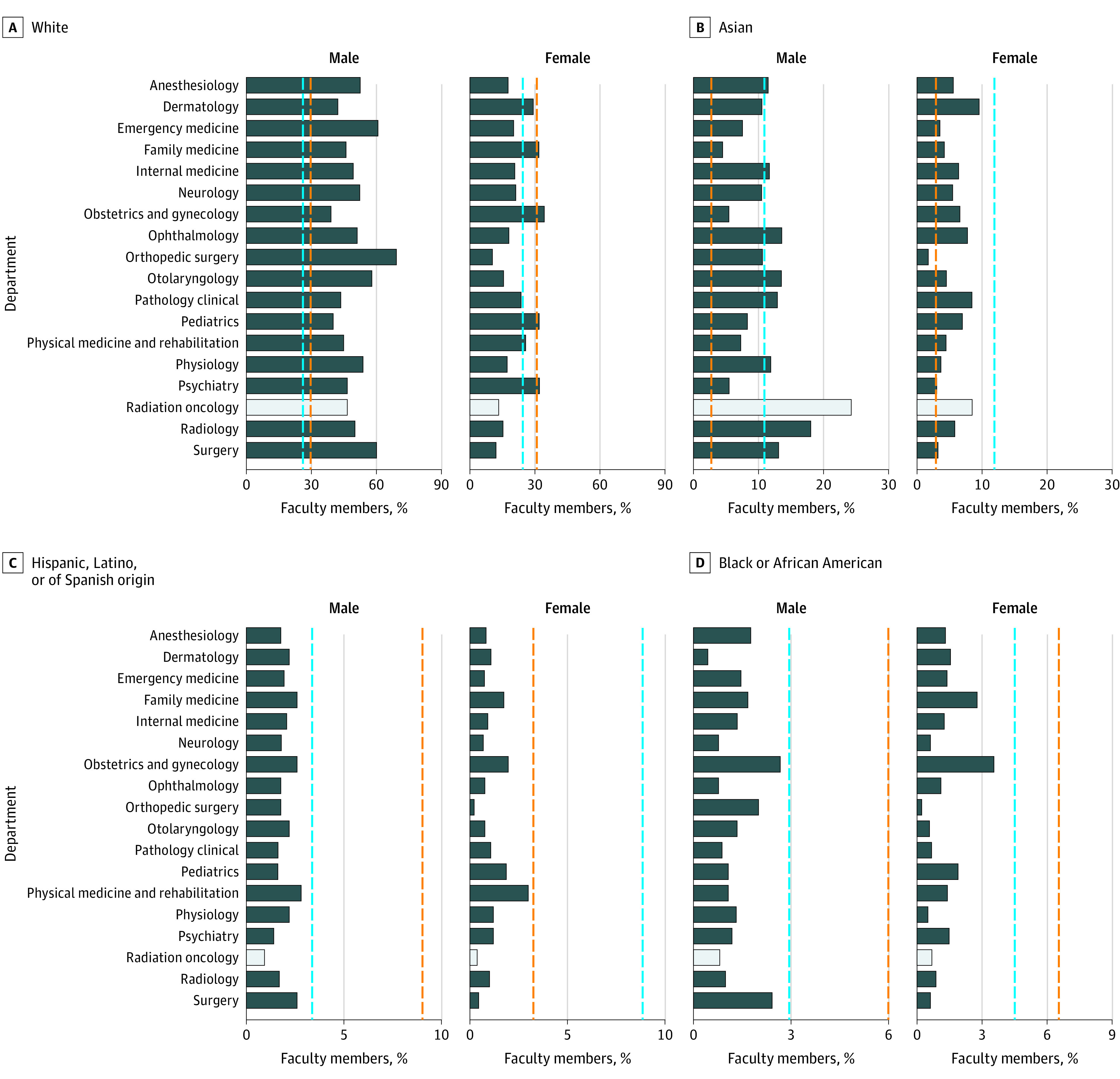
Demographics of Senior Faculty by Academic Medical Department in 2019 The bar graphs display the proportion of senior faculty in each US medical specialty by race, ethnicity, and sex in 2019. The light gray bar indicates the proportion of senior faculty in radiation oncology; the dashed orange line, the proportion of the general population compared with the proportion of senior faculty; the dashed blue line, the proportion of US medical students compared with the proportion of senior faculty during the 2019 academic year.

In 2019, there were 853 senior radiation oncology faculty (205 [24.03%] women). There were 279 Asian individuals [32.71%]; 13 Black individuals [1.52%]; 11 individuals with Hispanic, Latino or Spanish origin [1.30%]; 508 White individuals (59.55%); and 42 individuals with Native Hawaiian, other Pacific Islander, American Indian, Alaskan Native, unknown, multiracial, multiethnic, or other background (including 19 individuals with unknown race or ethnicity and 15 individuals with multiple race or ethnicity-non-Hispanic). Overall, there were 63 271 senior faculty in 2019 among 18 medical specialties studied (20 158 [31.86%] women). Among all specialties, there were 10 501 Asian individuals [16.60%]; 1712 Black individuals [2.71%]; 1923 individuals with Hispanic, Latino, or Spanish origin [3.04%]; 45 330 White individuals (71.64%); and 3805 individuals with unknown or multiracial or multiethnic background [6.01%]. Among 18 medical specialties (Figure 1), radiation oncology had the highest proportion of senior faculty who were Asian men (207 individuals [24.27%]) and 12th highest proportion of non-Hispanic White men (395 individuals [46.31%]), while having the fourth lowest and lowest proportion of non-Hispanic Black men (7 individuals [0.82%]) and Hispanic men (8 individuals [0.94%]), respectively. Radiation oncology had the second highest proportion of senior faculty who were Asian women (72 individuals [8.44%]), but second, third, and seventh lowest proportions of Hispanic women (3 individuals [0.35%]), non-Hispanic White women (113 individuals [13.25%]), and non-Hispanic Black women (6 individuals [0.70%]), respectively.

**Figure 2.**
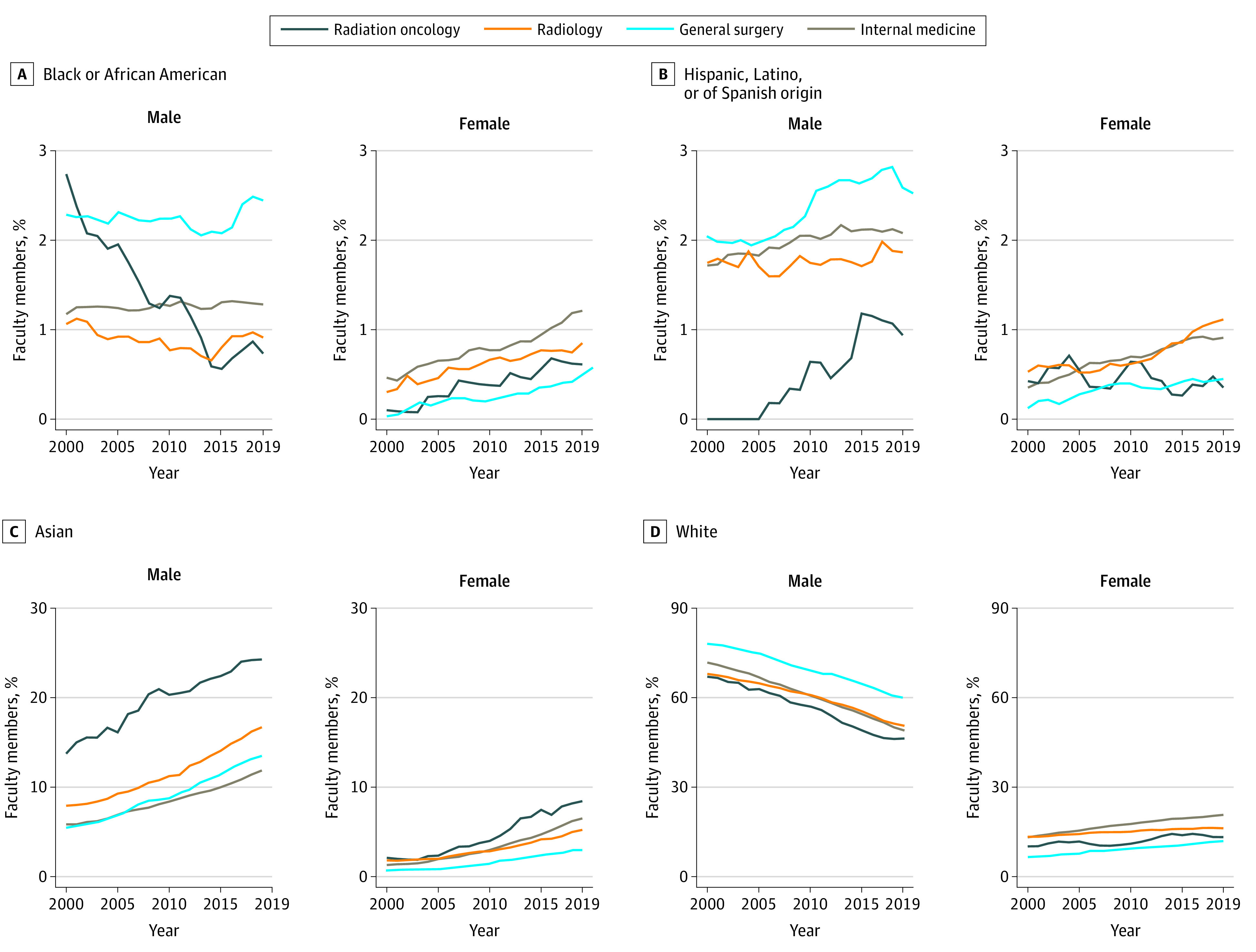
Demographic Changes in Senior Faculty The graphs display the proportion of senior faculty by sex, race, and ethnicity from 2000 to 2019 in radiation oncology, radiology, general surgery, and internal medicine.

Overall, small numbers of non-Hispanic Black and Hispanic male and female senior faculty within radiation oncology precluded analysis of changes in these groups from 2000 to 2019 (Figure 2). However, the proportion of non-Hispanic White male senior faculty in radiation oncology decreased from 317 of 466 individuals (68.03%) in 2000 to 395 of 834 individuals (47.36%) in 2019 (*P* < .001), while the proportion of Asian male senior faculty increased from 65 individuals (13.95%) to 207 individuals (24.82%) (*P* < .001). The proportion of non-Hispanic White female senior faculty significantly increased, from 48 individuals (10.30%) to 113 individuals (13.55%) (*P* < .001), as did the proportion of Asian female senior faculty, from 10 individuals (2.15%) to 72 individuals (8.63%) (*P* < .001). Proportions of senior faculty who were members of underrepresented minority groups did not significantly change from 2000 (20 of 402 men [4.98%]; 6 of 64 women [9.38%]) to 2019 (31 of 648 men [4.78%]; 15 of 279 women [5.38%]). There were 2-fold as many male as female radiation oncology senior faculty who were members of underrepresented minority groups in 2019.

In radiation oncology, the proportion of female senior faculty increased by 0.69% (95% CI, 0.59%-0.68%) per year, a significantly higher rate compared with general surgery (0.48% [95% CI, 0.43%-0.52%] per year; *P* < .001) and radiology (0.44% [95% CI, 0.39%-0.49%] per year; *P* < .001). Among faculty with known race or ethnicity, there were significant increases from 2000 to 2019 in male and female senior faculty who were members of underrepresented minority groups in internal medicine (559 of 9416 men [5.94%] vs 1177 of 11 828 men [9.95%]; *P* < .001; 145 of 1801 women [8.05%] vs 648 of 5414 women [11.97%]; *P* < .001), general surgery (313 of 4225 men [7.41%] vs 619 of 6146 men [10.07%]; *P* < .001; 21 of 366 women [5.74%] vs 131 of 1264 women [10.36%]; *P* = .002), and radiology (128 of 1996 men [6.41%] vs 199 of 2435 men [8.17%]; *P* < .001; 38 of 412 women [9.22%] vs 102 of 815 women [12.52%]; *P* = .01) but not in radiation oncology.

## Discussion

Addressing physician workforce diversity calls for reevaluation of the entire pipeline from early education to faculty promotion.^3^ Few studies, to our knowledge, have examined the combined associations of race, ethnicity, and sex with academic rank among medical faculty, although data tracking changes in groups with intersecting characteristics identifies specific areas of unmet need. Intersectionality also captures multiple demographic categories that reflect individual experiences with racism and sexism.^4^ For example, our cross-sectional study found that multiple departments lack adequate numbers of representative female senior faculty who are members of underrepresented minority groups, while radiation oncology has some of the lowest numbers. This study was limited by lack of information on who, when, or why individuals were hired, promoted, or left academia. However, programs that facilitate mentoring, networking, and leadership opportunities may be associated with improved representation in radiation oncology, as may substantive policies that address systemic exclusion of women, members of underrepresented minority groups, and faculty facing combined barriers related to race, ethnicity, and sex.^3,4,5,6^
